# Supplementary material for: The interactome of intact mitochondria by cross-linking mass spectrometry provides evidence for coexisting respiratory supercomplexes
Source: Mol Cell Proteomics. 2017 Dec 8;17(2):216–32. doi: 10.1074/mcp.RA117.000470 (PMC5795388; doi:10.1074/mcp.RA117.000470)
Supplement: Supplemental Data [file supp_RA117.000470_133922_0_supp_23978_fzffwf.zip › spectra_annotation/mito_spectra_annotation/265-1-4-1-3-1.pdf]

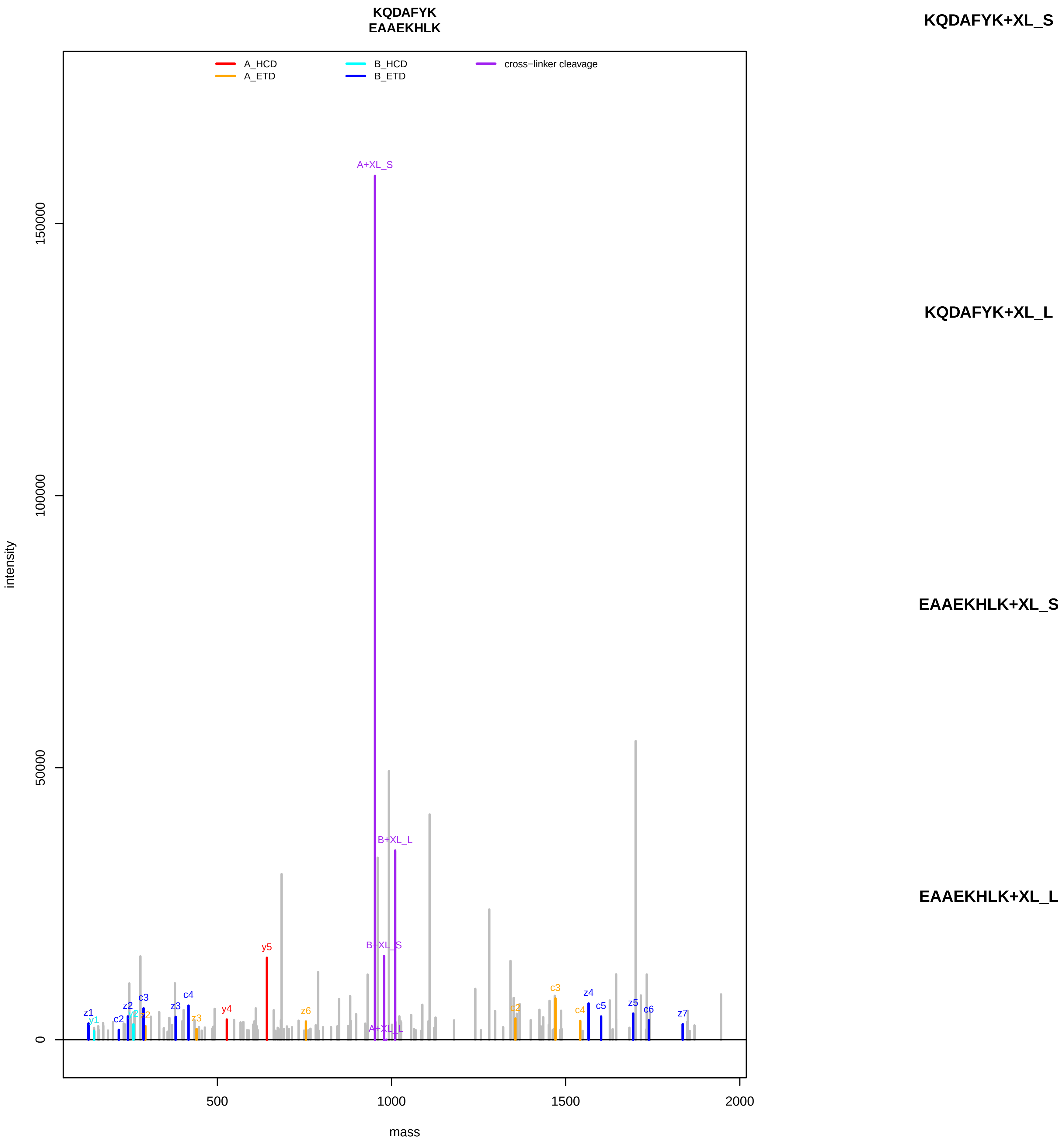
KQDAFYK
EAAEKHLK
A_HCD
A_ETD
B_HCD
B_ETD
cross−linker cleavage
A+XL_S
B+XL_L
B+XL_S
A+XL_L
y5
y4
z1
y1
c2
z2
y2
z2
c3
z3
c4
z3
z6
c2
c3
c4
z4
c5
z5
c6
z7
intensity
mass
0
50000
100000
150000
500
1000
1500
2000
KQDAFYK+XL_S
KQDAFYK+XL_L
EAAEKHLK+XL_S
EAAEKHLK+XL_L
